# Effects of Organizational Atmosphere and Organizational Practice on Knowledge, Attitude, and Practice Toward Diffusion and Utilization of Hepatic Contrast-Enhanced Ultrasound Among Physicians

**DOI:** 10.3389/fpubh.2022.778253

**Published:** 2022-03-14

**Authors:** Junhong Lu, Qingwen Deng, Wenbin Liu

**Affiliations:** Department of Health Management, School of Public Health, Fujian Medical University, Fuzhou, China

**Keywords:** technology diffusion, medical alliance, knowledge-attitude-practice, contrast-enhanced ultrasound, organizational readiness for change, theory of rational action

## Abstract

**Background:**

Promoting technology diffusion and utilization is a key measure to address the great disparity in technical capacity within integrated health systems. However, even the effectiveness and appropriateness regarding technology has been widely recognized, its diffusion and utilization are still stagnant. The mechanisms that influence the technology from being recognized to being widely applied in practice remain largely unknown.

**Purpose:**

Taking hepatic contrast-enhanced ultrasound (CEUS) as an example, this study aimed to investigate the comprehensive influencing mechanism of organizational atmosphere and organizational practice on the knowledge, attitude, and practice toward diffusion and utilization of hepatic CEUS in the medical alliance.

**Methods:**

Based on the integration of organizational ready for change (ORC) and knowledge-attitude-practice (KAP), a structured questionnaire was developed. A multistage random sampling method was applied to investigate physicians who directly use CEUS working at the liver disease-related departments of sampled health institutions. Structural equation modeling (SEM) was used to verify the proposed hypotheses, and determine the relationship between the factors.

**Results:**

In total, 292 physicians were included. SEM results demonstrated that knowledge influenced both attitude and practice, while attitude positively predicted practice. Organizational practice and organizational atmosphere associated positively with each other. Organizational atmosphere positively affected the physicians' attitude toward CEUS diffusion and utilization (β = 0.425, *p* < 0.001), while organizational practice positively affected corresponding knowledge (β = 0.423, *p* < 0.001) and practice (β = 0.275, *p* < 0.001). Additionally, there was a partial mediating effect between organizational practice and physicians' CEUS diffusion and utilization behavior.

**Conclusion:**

By verifying the influencing mechanism of organizational atmosphere and organizational practice on the physicians' KAP of hepatic CEUS diffusion and utilization, this study benefit tailoring strategies for promoting technology diffusion and utilization within medical alliance. It is recommended to develop an organizational atmosphere of advocating technology innovation, establish organizational support mechanism (SM) with multiple concrete supporting countermeasures, and so on.

## Introduction

Although integrated health systems have been widely promoted around the world (1), the great disparity in technical capacity between primary health institutions or county hospitals and large urban hospitals has been identified as a key concern. In particular, the delays or limitations in the diffusion and utilization of effective and appropriate technologies within medical alliance have greatly halted the improvement of the technical capacity of lower-level health institutions, which not only restricts the due role of specific technologies in a wider range and weakens the overall service capacity of the health system, but also exerts extremely adverse effects on public health (2).

Taking hepatic contrast-enhanced ultrasound (CEUS), for example, as a rapidly developing ultrasound examination technology with high sensitivity and specificity for the early detection and diagnosis of liver cancer, expanding the implementation of hepatic CEUS is becoming increasingly prominent, especially in the grim context of liver cancer prevention and control (3). However, with its effectiveness and appropriateness have been well proven and widely recognized (4), the diffusion and utilization of hepatic CEUS among physicians are still not well established, even in cases that many member institutions of medical alliance are qualified or sufficiently competent to provide regarding technical services (5–7). This fact reminds us that there is a complicated process for a certain technology from the general acceptance to its widespread use in practice. To steadily promote the diffusion and utilization of the technology, more effort should be invested to deeply investigate the comprehensive influencing mechanism underlying this process.

As stated by knowledge-attitude-practice (KAP) theory, the process of human behavior change can be divided into three steps, namely, acquiring knowledge, generating attitudes/beliefs, and forming practice/behaviors (8). The cognitive change caused by knowledge acquisition does not necessarily lead to behavior change; it should first lead to a change in perception, and then change people's behavior through the change in perception (9). Similarly, physicians' knowledge or the cognition of hepatic CEUS needs to lead to the extensive conceptual changes in the aspect of the early detection and diagnosis of liver cancer, and then gradually change the technology utilization behavior of hepatic CEUS through practical activities.

With respect to the potential influencing factors underlying the KAP process, some classical theoretical models, such as the theory of planning behavior (TPB), theory of rational action (TRA), and technology acceptance model (TAM), have provided clues alluding to some factors at the technical and individual levels (10, 11). Many previous empirical studies on technology diffusion and utilization behavior have focused on the technology itself and the groups, such as medical staff who directly use the technology and have verified the association between the final technology utilization behavior and individual-level (or technology-level) factors (11–14). However, even having been proposed by organizational ready for change (ORC) theory that some organizational features (organizational culture [OC], structures, resource endowments, etc.) may create a more receptive context for technology innovation and change (15–17), it has not been subjected to extensive empirical research, which makes the comprehensive influencing mechanism of organizational factors on certain health technology utilization behavior remain largely unknown. Especially in the context of medical alliance with many health institutions and medical staff, to further expand the use of appropriate and effective technologies, the role of organizational atmosphere and organizational practice in the KAP process of certain technology innovation implementation still needs to be further explored.

Therefore, this study intends to take hepatic CEUS as an example and integrate the ORC and KAP theories to investigate the comprehensive influencing mechanism of organizational atmosphere and organizational practice on technology diffusion and utilization in the medical alliance. The results of this study will be not only help to clarify the influence path of organizational-level factors on the knowledge, attitude, and finally the utilization behavior of specific technologies in the integrated healthcare system, but also provide practical guidance for promoting the diffusion and utilization of corresponding technologies in the context of the medical alliance and improving the service capacity of the overall health system.

## Materials and Methods

### Theoretical Framework

**Figure 1:**
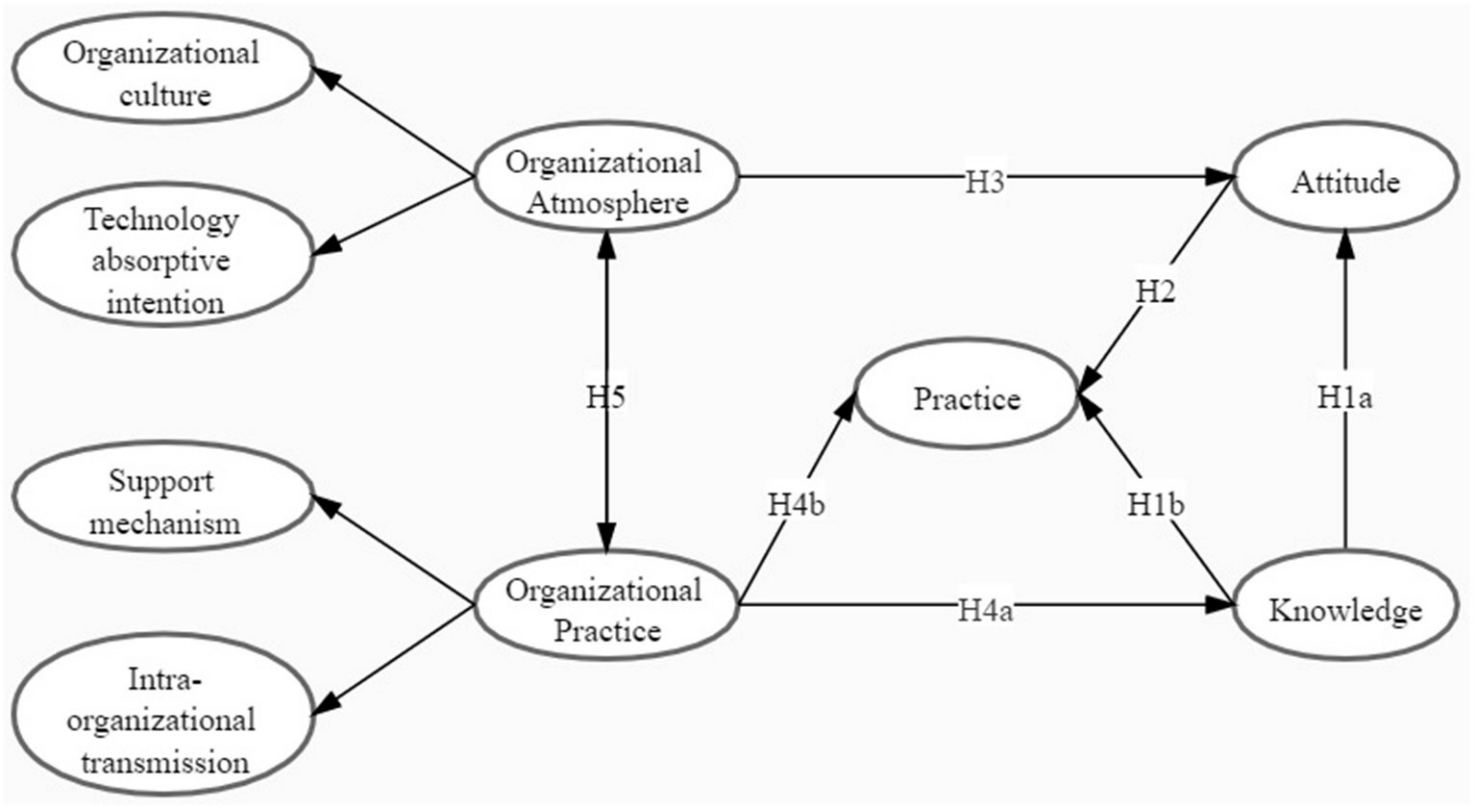
The theoretical model.

The theoretical model of this study was derived from the integration of ORC and KAP, which took seven elements into account, namely, OC, technology absorptive intention (TAI), support mechanism (SM), intra-organizational transmission, knowledge, attitude, and practice. Among them, an OC is a measure of the hospital's overall tendency and the acceptance of technological innovation as well as the degree of exchange and the sharing of technical knowledge or information (9, 18). If medical institutions attach importance to reform and advocate innovation, a favorable organizational atmosphere will be created for the diffusion and utilization of technology. Technology absorptive intention reflects the willingness of hospitals to collect information related to innovative technology so as to better absorb and introduce it into hospitals. A higher willingness to absorb technology can promote the formation of technology diffusion atmosphere and conditions in a wide rang of hospitals, and is conducive to technology utilization by the institutions themselves (19). Support mechanism is the human and material support provided by hospitals to promote the use of health technology, which reflects the positive willingness of senior leaders to widely use corresponding technology in hospitals, and is generally reflected in the aspects of budget, full-time personnel, and information feedback channels (20). Intra-organization transmission is a series of activities that have been carried out within the hospital to promote the use of corresponding technology, such as the provision of relevant training before and during the middle stage, information dissemination among members, and feedback summary in the later stage (21). Based on above understandings, the theoretical hypothesis model of this study is proposed in Figure 1, and the corresponding hypotheses (H) are as follows:

H1a: Physicians' knowledge of hepatic CEUS is influenced by their attitude;
H1b: Physicians' knowledge of hepatic CEUS is influenced by their practice;
H2: Physicians' attitude of hepatic CEUS is influenced by their practice;
H3: Physicians' attitude of hepatic CEUS is influenced by organizational atmosphere;
H4a: Physicians' knowledge of hepatic CEUS is influenced by organizational practice;
H4b: Physicians' practice of hepatic CEUS is influenced by organizational practice;
H5: There was a positive association between organizational atmosphere and organizational practice.

### Measurement

The measurement dimensions and specific measurement variables of this study were formed based on the theoretical model initially constructed and the scale content designed by previous studies. The scale consists of the following 3 parts:

The first part was the basic information of physicians, including 5 socio-demographic variables, such as gender, education level, professional title, administration position, and years of practice. The education level was divided into college and below, undergraduate, master's degree or above. The professional title of physicians included three levels, such as junior, intermediate, and senior. A physician who is also a director or deputy director of hospital administration can be considered to have an administrative position, otherwise there is no administrative position. The years of practice were classified into five groups: <5, 15~10, 11~15, 16~20, and >20 years.

The second part was the component to measure the current situation of physicians' KAP on the diffusion and utilization of hepatic CEUS, such as 8 questions from 3 dimensions. Considering the design and length of the initial questionnaire, “Knowledge” measures the physicians' awareness of the clinical principles of CEUS in the diagnosis of early liver cancer and their understanding of its advantages and disadvantages. “Attitude” is mainly measured from the perspective of whether the use of CEUS technology conforms to the personal values of physicians. “Practice” measures the physicians' formal use of CEUS techniques. “Knowledge” and “Practice” items were set according to the corresponding items in Beyer (22) and Estabrooks (23) scales. The variable of “Attitude” was measured by three items derived from the TPB scale.

The third part with 4 dimensions was about the organizational atmosphere and organizational practice on hepatic CEUS diffusion and utilization in the hospital where physicians work. Organizational atmosphere includes the dimensions of “OC” and “TAI.” Organizational practice includes the dimensions of “SM” and “intra-organization transmission” (IOT). The measurement of OC, TAI, SM, and intra-organizational transmission refers to the research of Helfrich et al. (19) and Mccormack et al. (21). All the items were measured using a 5-point Likert scale ranging from “strongly disagree” (1) through “neutral” (3) to “strongly agree” (5).

### Sample and Procedure

A cross-sectional questionnaire survey was conducted from February to August 2019 using a multi-stage random sampling method. First, taking the average incidence of liver cancer in China as the boundary (the incidence of liver cancer in China in 2015 was 26.92/100,000) (24), Fujian Province with a high incidence of liver cancer (31.70/100,000) (25), and Jiangxi Province with a low incidence of liver cancer (23.80/100,000) (26) were randomly selected. Second, all medical alliances in each province were listed as the sampling frame, and two medical alliances were randomly selected in each province. Third, in addition to investigate leading medical institutions, each medical alliance also randomly selected other medical institutions within the group at a proportion of 50%. Finally, the corresponding physicians from each sampled hospital who met the following inclusion criteria were selected as research subjects: physicians from departments related to liver disease diagnosis and treatment (such as, Hepatology, Liver surgery, Oncology, Gastroenterology, Infection, Radiotherapy, Intervention, and Ultrasound); knowing something about contrast-enhanced ultrasound technology. (In the questionnaire entitled “Do you know anything about the application of contrast-enhanced ultrasound in the early diagnosis of liver cancer,” subjects with “do not know at all” were excluded).

Each medical alliance was expected to investigate 5–8 medical institutions and 20–30 medical institutions will be included from the four medical alliances in the two provinces. Since 10–20 physicians will be surveyed in each institution, at least 200 physicians will be involved in total, which can fully meet the basic requirement that the sample size should be set at least five times survey questions (27). With the support of selected health institutions, each round for filling out the questionnaire was accompanied by a trained coordinator to introduce the study purpose.

### Data Analysis

First, to determine whether the questionnaire was acceptable, the reliability of the questionnaire was evaluated by calculating the Cronbach's alpha coefficient, and its validity was assessed by factor analysis. Second, the socio-demographic characteristics and the score of each item in the scale were analyzed descriptively. Finally, structural equation modeling (SEM) was conducted to validate the hypotheses and determine the mechanism among organizational atmosphere, organizational practice, and the KAP of physicians' hepatic CEUS diffusion and utilization. Chi-square/df, Goodness-of-Fit Index (GFI), Adjust the Goodness-of-Fit Index (AGFI), root-mean-square error of approximation (RMSEA), and Comparative Fit Index (CFI) were used for checking the fitness of the model. Data analyses were performed using SPSS 21.0 and AMOS 17.1 software programs. In all reported analysis, *p* < 0.05 was considered statistically significant.

## Results

### Socio-Demographic Characteristics of Participants

A total of 292 physicians were included in this study, with 65.1% of them being men. Most of the participants have bachelor's degree or above, of which 40.4% have a master's degree or above. The proportions of junior, middle, and senior professional titles were 37.0, 39.7, and 23.3%, respectively. Most physicians have no administration positions, and 32.2% of them had 5–10 years of practice. The participants' socio-demographic information is demonstrated in Table 1.

**Table 1:** Socio-demographic characteristics of the 292 participants.

**Characteristic**	**Frequency (*n*)**	**Percentage (%)**
**Gender**		
Male	190	65.1
Female	102	34.9
**Education level**		
College and below	18	6.2
Undergraduate	156	53.4
Master's degree or above	118	40.4
**Professional titles**		
Junior	108	37.0
Intermediate	116	39.7
Senior	68	23.3
**Administration position**		
No	242	82.9
Yes	50	17.1
**Years of practice**		
<5 years	74	25.3
5–10 years	94	32.2
11–20 years	88	30.1
21–30 years	30	10.3
>30 years	6	2.1

### Reliability and Validity

Table 2 reports Cronbach's alpha, composite reliability (CR), and the average variance extracted (AVE). The Cronbach's alpha of 5 dimensions and the entire questionnaire were all greater than the recommended 0.7 threshold (28), ranging from 0.818 to 0.949, indicating good internal consistency. Besides, all factor loading values of items were above the acceptability value of 0.5 (29). Moreover, the CR scores and AVE values of all constructs were above the recommended value of 0.7 (30) and 0.5 (31), respectively, which indicated a good convergent validity.

**Table 2:** Results of confirmatory factor analysis.

**Dimension**	**Item**	**Factor loading**	**AVE**	**CR**	**Cronbach's Alpha**
OA	OC	0.862	0.804	0.954	0.886
	TAI	0.930			
OP	SM	0.904	0.795	0.950	0.881
	OIS	0.879			
K	K1	0.895	0.878	0.983	0.933
	K2	0.977			
A	A1	0.933	0.862	0.978	0.949
	A2	0.908			
	A3	0.944			
P	P1	0.852	0.820	0.962	0.930
	P2	0.929			
	P3	0.934			
The whole questionnaire					0.818

*CR, Composite Reliability; AVE, Average Extracted Variance; OA, organizational atmosphere; and OP, organizational practice*.

### Score Situation

As shown in Table 3, the skewness value of each item was between −3 and 3, while the kurtosis value was <7, indicating that the data of each item conforms to the normal distribution. The mean score for the items in the dimension of organizational atmosphere was higher than that in the dimension of organizational practice. In addition, the mean score for the items in the dimension of attitude were higher than that in the dimension of knowledge and practice, respectively.

**Table 3:** Organizational atmosphere, organizational practice, and physicians' CEUS diffusion and utilization of knowledge, attitude, and practice (KAP) scores.

**Dimension**	**Item**	**Mean**	**Standard deviation**	**P25**	**P75**	**Skewness**	**Kurtosis**
OA							
	OC	4.070	1.072	3.667	5.000	−1.336	1.469
	TAI	3.796	1.184	3.000	5.000	−0.965	0.188
OP							
	SM	2.683	1.218	1.667	3.667	0.047	−1.026
	IOT	2.501	1.341	1.000	3.667	0.344	−1.109
KAP							
	K1	2.781	1.241	2.000	4.000	−0.001	−0.994
	K2	2.753	1.249	2.000	4.000	0.008	−1.065
	A1	4.240	0.872	4.000	5.000	−1.238	1.801
	A2	4.271	0.873	4.000	5.000	−1.274	1.837
	A3	4.223	0.859	4.000	5.000	−1.069	1.245
	P1	1.952	1.305	1.000	3.000	1.127	−0.019
	P2	2.233	1.457	1.000	3.750	0.682	−1.057
	P3	2.092	1.422	1.000	3.000	0.897	−0.697

*OA, organizational atmosphere; and OP, organizational practice*.

### SEM Analysis and Hypothesis Testing

**Figure 2:**
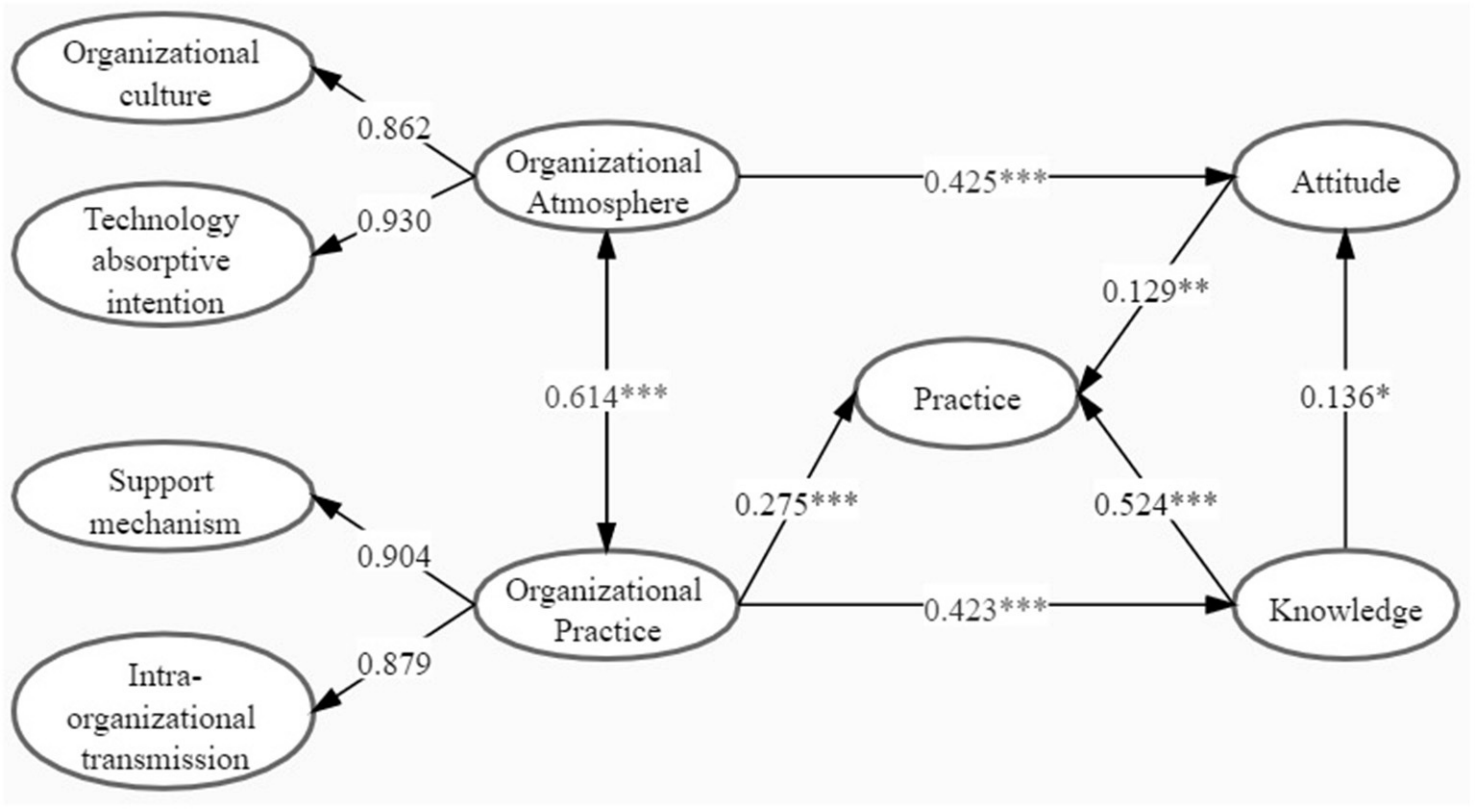
A model of organizational atmosphere and organizational practice on knowledge, attitude, and practice (KAP) toward the diffusion and utilization of hepatic CEUS among physicians (*p < 0.05; **p < 0.01); and ***p < 0.001.

A favorable fitness of data into the theoretical framework was found: χ^2^/*df* = 2.785, GFI = 0.931, AGFI = 0.885, CFI = 0.973, and RMSEA = 0.078 (<0.08). The final structural model with the standardized estimates is presented in Figure 2 and Table 4.

**Table 4:** The results of standardized direct, indirect, and total effects.

**Paths**	**Direct effects (Path coefficients)**	**Indirect** **effects**	**Total effects**
Knowledge → Attitude	0.136^*^	0	0.136^*^
Knowledge → Practice	0.524^*^	0.018^*^	0.542^**^
Attitude → Practice	0.129^*^	0	0.129^*^
Organizational atmosphere → Attitude	0.425^**^	0	0.425^**^
Organizational atmosphere → Practice	0	0.055^*^	0.055^*^
Organizational practice → Knowledge	0.423^*^	0	0.423^*^
Organizational practice → Attitude	0	0.057^*^	0.057^*^
Organizational practice → Practice	0.275^*^	0.229^**^	0.504^*^

* *p < 0.05;*

** *p < 0.01*.

The hypothesis test results showed that the knowledge of physicians on the diffusion and utilization of hepatic CEUS had a positive effect on their attitude (β = 0.136, *p* < 0.05) and practice (β = 0.524, *p* < 0.001). The significant effect was also detected between the physicians' attitude and their practice on the diffusion and utilization of hepatic CEUS (β = 0.129, *p* < 0.01). Additionally, organization practice has a significant impact on physicians' knowledge (β = 0.423, *p* < 0.001) and practice (β = 0.275, *p* < 0.001) of hepatic CEUS diffusion and utilization, while organizational atmosphere has a positive effect on physicians' attitude in hepatic CEUS diffusion and utilization (β = 0.425, *p* < 0.001). Positive association was demonstrated between organizational practice and organizational atmosphere (β = 0.614, *p* < 0.001). All seven hypotheses have passed the verification. In addition, calculated by Bootstrap test, the 95% *CI* for the impact of organizational practice on physicians' hepatic CEUS diffusion and utilization practice is 0.342–0.605, excluding 0, indicating that there was a mediating effect. Besides, the value of mediating effect was 0.229 (*p* < 0.01), which accounted for 45.44% of the total effect.

## Discussion

Under the current context of limited diffusion and the utilization of appropriate health technologies within an integrated health system (6, 32), it is essential to clarify the influencing path of organizational factors on the physicians' KAP of diffusion and utilization of corresponding technology, especially when the comprehensive influencing mechanism of organizational factors remain largely unknown. By taking hepatic CEUS as an example, this study integrated ORC and KAP theories to develop a hypothesized model, and further applied SEM to verify the influencing mechanism of organizational atmosphere and organizational practice on the physicians' KAP of diffusion and utilization of hepatic CEUS. The results of this study will benefit further diffusion and utilization of CEUS and other appropriate health technology within the integrated health systems.

On the one hand, some hypotheses respectively proposed on the basis of KAP and ORC were confirmed in this study. Consistent with KAP hypotheses and previous empirical research (33), this study showed that the physicians' knowledge of hepatic CEUS positively influences their attitude and practice toward corresponding technology utilization. Additionally, significant influence was detected from physicians' attitude to their technology utilization behavior. These results reminded us that some efforts, such as transmitting the knowledge of certain technologies and transforming the belief of corresponding technologies utilization, should be made previously to promote the occurrence of technology utilization behavior (9). Similarly, this study reported significant positive association relationship between organizational atmosphere and organizational practice, which was in line with ORC theory. It revealed that organizational atmosphere and organizational practice complement and promote each other. It is embodied in CEUS and other corresponding health technologies that to obtain financial support, personnel support, information support, training and learning, information communication and other aspects of organizational practice, normative OC and strong willingness to share technology are needed as support. Conversely, if the hospital establishes an effective SM and is willing to carry out systematic training and the learning of appropriate health technology practices, it can further promote the hospital to advocate technological innovation, continuously learn and absorb new technologies, and share technological knowledge and information with other medical institutions. Thus, in the process of preparing for the diffusion of technological innovation in hospitals, the importance between organizational atmosphere and organizational practice should be highlighted.

On the other hand, with respect to the impacts of organizational determinants on the factors at the individual level, this study demonstrated that organizational practice could positively predict the knowledge and practice of physicians in the diffusion and utilization of hepatic CEUS, and play a mediating role in their utilization behavior through knowledge. Besides, the significant direct impact of organizational atmosphere was detected on the physicians' attitude toward hepatic CEUS utilization.

In line with the findings of previous research that organizational practice including some concrete SM can directly motivate individual behavior (34). In addition, this study reported that physicians were more inclined to use hepatic CEUS under appropriate situation if they gained more organizational support for CEUS adoption. The underlying mechanism may be that organizational practice can act as an organizational assurance and implementation support agency by providing more funding support, delivering more updating information, and setting up innovation promoting department, which will subsequently stimulate the physicians' consciousness and subjective initiative of hepatic CEUS adoption and utilization (20, 35). In the mode of medical alliance in China, the relatively common loose mode of the medical alliance fails to sink effectively in human resources allocation and health technology, which is not conducive to the diffusion and utilization of technology (36). On the other hand, the compact medical alliance manages people and property uniformly, encourages physicians to provide medical service guidance to grass-roots institutions (36), further sinks and integrates high-quality medical resources. Additionally, some member institutions in compact medical alliance have even invited experts from superior institutions to hold salons and lectures to share their experiences and insights in CEUS utilization in clinical practice. This will not only improve the physicians' knowledge of CEUS and enhance their own ability, but also drive more physicians to participate in the practice of the diffusion and utilization of corresponding technology, which may be the plausible reason of organizational practice's significant indirect impact on their utilization behavior through knowledge.

Since organizational atmosphere is often considered as an invisible “public opinion environment” recognized and accepted by the public (37), it is not hard to understand that it will inevitably affect physicians' attitude toward the diffusion and utilization of corresponding technology. In addition to the significant impact of attitude on the final utilization practice as mentioned above, this study confirmed organizational atmosphere's significant indirect influence on physicians' hepatic CEUS utilization practice through the physicians' attitude. These results highlighted the importance of developing a good organizational atmosphere suitable for the diffusion and utilization of technology or innovation (38). Combined with the practice mode of the medical alliance in China, the atmosphere of health technology diffusion in the loose medical organization has not formed the common value of technology diffusion compared with the compact medical alliance (36), which fails to unite, motivate, and regulate the behavior of the hospital members. To be more specific, it is recommended that the common value of advocating technological innovation should be established on the basis of constructing close medical alliance. Especially for the medical alliance of liver disease, it is of vital importance of reaching a consensus within the medical alliance to encourage early diagnosis and early treatment for patients with liver cancer even those at a high risk of liver cancer. On this basis, the organizational values that actively advocate the innovation in the screening and diagnostic technology would be capable to develop, which will further improve the organizational readiness for the diffusion and utilization of corresponding technologies at the ideological level (35).

However, it is noteworthy that the organizational atmosphere cannot directly drive the change of physicians' hepatic CEUS adoption and utilization. The plausible reason may be that organizational atmosphere representing the hospital's mental readiness and preference for certain technology innovation is not the organizational practice, which includes some concrete supporting measures to directly promote the advent of individual behavior (39). Additionally, organizational atmosphere does not completely replace or represent the individual physicians' attitude or intention toward corresponding technology use, which are often deemed as important predictors of utilization behavior in classical TPB (40). This result implied that the practical effect of technology diffusion would be limited if merely taking the countermeasure for improving organizational atmosphere without any concrete organizational SM. According to the significant association between organizational atmosphere and SM as confirmed above, it would be wise to simultaneously provide some concrete supporting measures (such as, providing financial support, establishing full-time departments or personnel, and establishing information communication channels) to promote new technology diffusion and utilization while creating an organizational atmosphere to encourage technological innovation.

This study was strengthened by some features. First, the study was developed by integrating the strengths of previous ORC and KAP theories, which greatly expand the scope of theoretical utilization and pertinently bridge the knowledge gap of the influencing mechanism of organizational factors on the physicians' KAP of diffusion and utilization of appropriate health technologies. Second, the research results will not only contribute to promoting the diffusion and utilization of hepatic CEUS to a wider scope at certain institutions, but also provide insight for expanding the use of other appropriate health technologies within an integrated healthcare system. Third, the sampled hospitals came from Fujian and Jiangxi Provinces respectively with a high- and low-liver cancer incidence rate, which would improve the representativeness of the sample. Fourth, this study applied SEM to simultaneously determine the influence of several factors on the diffusion and utilization of certain technology (outcome variable), as well as their internal interactions, which made the result prediction more accurate and robust. However, there are also some limitations to this study. First, due to the effect of social desirability and personal subjectivity, the surveyed physicians may tend to make more positive evaluation of their own and hospital performance than the actual situation, which leads to the overestimation of hospital atmosphere, practice, and physicians' KAP. Second, due to the structural design and length limit of the questionnaire, it lacks objective indicators to measure the professional knowledge of physicians. Finally, further research can take more relevant factors into considerations, such as physician's expertise and other organization-level factors that affect the physicians' KAP of the diffusion and utilization of hepatic CEUS or other appropriate health technologies. Besides, we will further study the influence of corresponding variables in future research.

## Conclusion

By taking hepatic CEUS as an example, this study verified the influencing mechanism of organizational atmosphere and organizational practice on KAP toward the diffusion and utilization of corresponding technology. It is recommended to develop an organizational atmosphere of advocating technology innovation within the medical alliance and its member institutions. Additionally, to further expand the technology use to a wider scope, it also highlights the importance of establishing organizational SM. Some concrete supporting countermeasures are strongly recommended, such as sharing up-to-date information on corresponding technology or innovation, allocating special funds and personnel for technology innovation diffusion, and providing more training and guidance for newly introduced technologies.

## Data Availability Statement

The original contributions presented in the study are included in the article/Supplementary Material, further inquiries can be directed to the corresponding author.

## Ethics Statement

Ethical permission was granted for this study from the Ethics Committee of Fujian Medical University (No. 2017-17). This study was conducted under the Declaration of Helsinki. A formal letter of cooperation was written to the directors of each selected medical institution and permission was obtained. All participants were informed about the study purpose, participation in the study was voluntary, and all responses were anonymous.

## Author Contributions

WL designed and conducted the project, contributed to grasp the subject, and revised the manuscript. JL carried out the data analysis and drafted the manuscript. WL and QD developed the questionnaire. All authors read and approved the manuscript before submission.

## Funding

This research was supported by the National Natural Science Foundation of China (Grant Number: 71704026) and the Distinguished Young Scientific Research Talents Plan in Universities of Fujian Province (Grant Number: 2018B030). The funders had no involvement in study design, data collection, statistical analysis, and manuscript writing.

## Conflict of Interest

The authors declare that the research was conducted in the absence of any commercial or financial relationships that could be construed as a potential conflict of interest.

## Publisher's Note

All claims expressed in this article are solely those of the authors and do not necessarily represent those of their affiliated organizations, or those of the publisher, the editors and the reviewers. Any product that may be evaluated in this article, or claim that may be made by its manufacturer, is not guaranteed or endorsed by the publisher.

## References

[1] Shevsky V Sheiman I. Problems of Creating an Integrated Health System. Pub Admin Issues (2013). pp. 24–47. Available online at: https://diabetesatlas.org/upload/resources/previous/files/8/IDF_DA_8e-EN-final.pdf (accessed June 20, 2021).

[2] Silva HP Viana AL. Health technology diffusion in developing countries: a case study of CT scanners in Brazil. Health Policy Plan. (2011) 26:385–94. 10.1093/heapol/czq076 21118866

[3] Zou XN. Epidemic trend, screening, and early detection and treatment of cancer in Chinese population. Cancer Biol Med. (2017) 14:50–9. 10.20892/j.issn.2095-3941.2016.0047 28443203 PMC5365185

[4] Strobel D Seitz K Blank W Schuler A Dietrich C von Herbay A. Contrast-enhanced ultrasound for the characterization of focal liver lesions-diagnostic accuracy in clinical practice (DEGUM multicenter trial). Ultraschall Med. (2008) 29:499–505. 10.1055/s-2008-1027806 19241506

[5] Yang X Wang X Zhang Y. The Contrast-Enhanced Ultrasound Information Technology in the Treatment of Liver Tumors. J Med Imag Health In. (2020) 10:2168–74. 10.1166/jmihi.2020.3148

[6] Coughlin JM Zang Y Terranella S Alex G Karush J Geissen N. Understanding barriers to lung cancer screening in primary care. J Thorac Dis. (2020) 12:2536–44. 10.21037/jtd.2020.03.66 32642161 PMC7330370

[7] Dulai GS Farmer MM Ganz PA Bernaards CA Qi K Dietrich AJ. Primary care provider perceptions of barriers to and facilitators of colorectal cancer screening in a managed care setting. Cancer. (2010) 100:1843–52. 10.1002/cncr.20209 15112264

[8] Twinamasiko N Olum R Gwokyalya AM Nakityo I Wasswa E Sserunjogi E. Assessing Knowledge, Attitudes and Practices Towards COVID-19 Public Health Preventive Measures Among Patients at Mulago National Referral Hospital. Risk Manag Healthc Policy. (2021) 20:221–30. 10.2147/RMHP.S287379 33505175 PMC7829119

[9] Wang J Chen L Yu M He J. Impact of knowledge, attitude, and practice (KAP)-based rehabilitation education on the KAP of patients with intervertebral disc herniation. Ann Palliat Med. (2020) 9:388–93. 10.21037/apm.2020.03.01 32233633

[10] Dewi TK Zein RA. Predicting Intention Perform Breast Self-Examination: Application of the Theory of Reasoned Action. Asian Pac J Cancer Prev. (2017) 26:2945–52. 10.22034/APJCP.2017.18.11.2945 29172263 PMC5773775

[11] Mustafa MH Ahmad MB Shaari ZH Jannat T. Integration of TAM, TPB, and TSR in understanding library user behavioral utilization intention of physical vs. E-book format. J Acad Libr. (2021) 47:102399. 10.1016/j.acalib.2021.102399

[12] Jongchul O Sung-Joon Y. Validation of Haptic Enabling Technology Acceptance Model (HE-TAM): Integration of IDT and TAM. Telemat Inform. (2014) 31:585–96. 10.1016/j.tele.2014.01.002

[13] Liu C. Understanding Electronic Commerce Adoption at Organizational Level: Literature Review of TOE Framework and DOI Theory. Int Journal Sci Bus. (2019) 3:179–95. Available online at: https://ijsab.com/wp-content/uploads/336.pdf

[14] Deng Q Liu W. Utilization of clinical practice guideline on antimicrobial in China: an exploratory survey on multilevel determinants. BMC Health Serv Res. (2020) 20:282. 10.1186/s12913-020-05171-z 32252756 PMC7137508

[15] Ly BA Labonté R Bourgeault IL. The beliefs of Senegal's physicians toward the use of telemedicine. Pan Afr Med J. (2019) 34:97. 10.11604/pamj.2019.34.97.20216 31934240 PMC6945665

[16] Askari M Tam JLYY Aarnoutse MF. Perceived effectiveness of clinical pathway software: A before-after study in the Netherlands. Int J Med Inform. (2020) 135:104052. 10.1016/j.ijmedinf.2019.104052 31865190

[17] Weiner BJ. A theory of organizational readiness for change. Implement Sci. (2009) 4:67. 10.1186/1748-5908-4-67 19840381 PMC2770024

[18] Schein EH. Organizational culture. Am Psychol. (1990) 45:109–19. 10.1037/0003-066X.45.2.109

[19] Helfrich CD Li YF Sharp ND Sales AE. Organizational readiness to change assessment (ORCA): development of an instrument based on the Promoting Action on Research in Health Services (PARIHS) framework. Implement Sci. (2009) 4:1–13. 10.1186/1748-5908-4-38 19594942 PMC2716295

[20] Gaddam SR. Timing and Assimilation of New Technology Adoption in Healthcare. J Health Med Inform. (2019) 10:2. Available online at:https://www.hilarispublisher.com/open-access/timing-and-assimilation-of-new-technology-adoption-in-healthcare.pdf

[21] Mccormack B Mccarthy G Wright J Slater P Coffey A. Development and testing of the Context Assessment Index (CAI). Worldviews Evid Based Nurs. (2009) 6:27–35. 10.1111/j.1741-6787.2008.00130.x 19207560

[22] Beyer JM. Research utilization: Bridging the gap between communities. J Manage Inquiry. (1997) 6:17–22. 10.1177/105649269761004

[23] Estabrooks CA. The conceptual structure of research utilization. Res Nurs Health. (1999) 22:203–16. 10.1002/(sici)1098-240x(199906)22:3<203::aid-nur3>3.0.co;2-9 10344701

[24] Zhang S Sun K Zheng R Zeng H Wang S Chen R. Cancer incidence and mortality in China, 2015. J Natl Cancer Center. (2021) 1:2–11. 10.1016/j.jncc.2020.12.001 PMC11256613 39036787

[25] Lin Y Zhou Y Ma J. Cancer incidence and mortality in Fujian cancer Registration areas from 2015 to 2017. China Cancer. (2021) 30:487–94. Chinese. 10.11735/j.issn.1004-0242.2021.07.A002

[26] Liu J Zhu LP Yang XL Xu Y Yan W Chen YY. Incidence, mortality and life lost of malignancies among residents living in areas covered by cancer registry in Jiangxi province, 2010-2017. Chinese J Pub Health. (2018) 34:1692–5. Chinese. 10.11847/zgggws1120543

[27] Gorusch RL. Factor Analysis. Hillsdale: Lawrence Erlbaum Associates (1983). p. 332.

[28] Nunnally JC. Psychometric Theory. 2nd ed. New York: McGraw-Hill (1978).

[29] Fornell C Larcker DF. Evaluating structural equation models with unobservable variables and measurement error. J Mark Res. (1981) 18:39–50. 10.1177/002224378101800313

[30] Gefen D Straub DW Boudreau MC. Structural equation modeling and regression: guidelines for research practice. Commun Assoc Inf Syst. (2000) 4:7. 10.17705/1CAIS.00407

[31] Bagozzi RP Yi Y. On the evaluation of structure equation models. J Acad Mark Sci. (1998) 16:74–94. 10.1007/BF02723327

[32] Uscher-Pines L Kahn JM. Barriers and facilitators to pediatric emergency telemedicine in the United States. Telemed J E Health. (2014) 20:990–6. 10.1089/tmj.2014.0015 25238565 PMC4229699

[33] Zhong BL Luo W Li HM Zhang QQ Liu XG Li WT. Knowledge, attitudes, and practices towards COVID-19 among Chinese residents during the rapid rise period of the COVID-19 outbreak: a quick online cross-sectional survey. Int J Biol Sci. (2020) 16:1745–52. 10.7150/ijbs.45221 32226294 PMC7098034

[34] Kaffashpoor A Shojaean A Toosi M. Effect of Perceived Organizational Support on Organizational Citizenship Behaviors with emphasis on the mediating role of Job Satisfaction. Iran J Nur Res. (2017) 12:42–9. 10.21859/ijnr-12017

[35] Williams I. Organizational readiness for innovation in health care: some lessons from the recent literature. Health Serv Manage Res. (2011) 24:213–8. 10.1258/hsmr.2011.011014 22040949

[36] Cai Y Wen C Tang L Liu P Xu Y Hu S. Exploration and Consideration of the Medical Alliance Modes. Iran J Public Health. (2018) 47:1160–5. Available online at: https://www.ncbi.nlm.nih.gov/pmc/articles/PMC6123575/30186788 PMC6123575

[37] Phankhong T Bakar LA Poespowidjojo D. The Mediating effect of Innovativeness on Innovation Strategy, Atmosphere, Culture and Organizational Performance: Proposed theoretical Framework. Int J Econ Res. (2017) 14:359–69. Available online at: http://repo.uum.edu.my/id/eprint/24491/1/The%20Mediating%20effect%20of%20Innovativeness%20on%20Innovation%20Strategy,%20Atmosphere,%20Culture%20and%20Organizational%20Performance%20Proposed%20theoretical%20Framework%20(Scopus).pdf

[38] Deng Q Lu J Zeng Z Zheng Y Liu W. Dynamics of Health Technology Diffusion in the Integrated Care System (DHTDICS): A Development and Validation Study in China. Risk Manag Healthc Policy. (2021) 14:331–44. 10.2147/RMHP.S293144 33536802 PMC7850575

[39] Liu C Liu C Wang D Zhang X. Intrinsic and external determinants of antibiotic prescribing: a multi-level path analysis of primary care prescriptions in Hubei, China. Antimicrob Resist Infect Control. (2019) 8:132. 10.1186/s13756-019-0592-5 31406571 PMC6686458

[40] Rantanen T Lehto P Vuorinen P Coco K. The adoption of care robots in home Care—a survey on the attitudes of Finnish home care personnel. J Clin Nurs. (2018) 27:1846–59. 10.1111/jocn.14355 29575204

