# Supplementary material for: Effects of Organizational Atmosphere and Organizational Practice on Knowledge, Attitude, and Practice Toward Diffusion and Utilization of Hepatic Contrast-Enhanced Ultrasound Among Physicians
Source: Front Public Health. 2022 Mar 14;10:778253. doi: 10.3389/fpubh.2022.778253 (PMC8964784; doi:10.3389/fpubh.2022.778253)
Supplement: Supplementary file 1 [file Data_Sheet_1.PDF]

The research questionnaire on the factors influencing the diffusion and utilization of hepatic contrast-enhanced ultrasound

Part 1. Personal Information Card

Notes. The following questions are some basic information about you, please circle the number or fill in the blank that best matches your real situation.

1. Gender:
   A. Male B. Female
2. Age: ________
3. Educational level:
   A. Junior college or below B. Bachelor C. Master D. Doctor
4. Professional title:
   A. Junior B. Intermediate C. Senior
5. Do you have administration positions:
   A. Yes B. No
6. Years in practice:
   A. <5 years B. 5~10 years C. 11~15years D. 16~20 years E. >20 years

Part 2. Physicians' knowledge, attitude and practice on the diffusion and utilization of contrast-enhanced ultrasound (CEUS)

Notes. There are 5 numbers (1, 2, 3, 4, 5) on the right side of each item, where "1" means "Strongly disagree", "2" means "Disagree", "3" means "Neutral", "4" means "Agree", and "5" means "Strongly agree". Please tick or circle the number that best fits your real feelings on the item.

Knowledge					
Knowledge of the clinical principle of CEUS in the diagnosis of early hepatocellular carcinoma	1	2	3	4	5
Understanding of the advantages and disadvantages of CEUS in the diagnosis of early hepatocellular carcinoma	1	2	3	4	5
Attitude					
I think it's a right thing to use CEUS for early diagnosis of hepatocellular carcinoma.	1	2	3	4	5
I think it's a wise choice to use CEUS for early diagnosis of hepatocellular carcinoma.	1	2	3	4	5
I think it's good for all to use CEUS for early diagnosis of hepatocellular carcinoma.	1	2	3	4	5
Utilization behavior					
In the past year, the probability that I use CEUS on all working days.	1	2	3	4	5
In the past year, the probability that I skillfully combine the CEUS results to make clinical diagnosis.	1	2	3	4	5
In the past year, the probability that I recommended further using CEUS to my peers.	1	2	3	4	5

Part 3. The scale of organizational norms

Notes. There are 5 numbers (1, 2, 3, 4, 5) on the right side of each item, where “1” means “Strongly disagree”, “2” means “Disagree”, “3” means “Neutral”, “4” means “Agree”, and “5” means “Strongly agree”. Please tick or circle the number that best fits your real feelings on the item.

Hospital culture					
The hospital advocates the technical innovation to improve the clinical outcomes for patients.	1	2	3	4	5
The hospital advocates continuous learning and absorption of new technologies.	1	2	3	4	5
The hospital advocates the exchange and sharing of clinical experience.	1	2	3	4	5
Technology absorptive intention					
When the CEUS test appeared, the hospital is willing to allocate staff to collect information.	1	2	3	4	5
When the CEUS test introduced, the hospital is willing to provide training for the staff.	1	2	3	4	5
When the CEUS test was adopted for clinical practice, the hospital is willing to promote its use more widely.	1	2	3	4	5
Support mechanisms					
Hospital provided funding support for the introduction of CEUS technology to carry out related clinical services.	1	2	3	4	5
Hospital have designated the department or personnel responsible for technology introduction and application in the hospital.	1	2	3	4	5
Information communication channels have been established by the hospital for timely feedback of problems.	1	2	3	4	5
Intra-Organizational Transmission					
The hospital has carried out systematic training and learning on the experience and skills of clinical application of CEUS.	1	2	3	4	5
Information on the clinical application of CEUS is disseminated and exchanged among hospital members.	1	2	3	4	5
The internal members of the hospital discussed the problems in the application of CEUS technology in the early diagnosis of liver cancer and summarized the experience.	1	2	3	4	5
